# Translational Potential of Stem Cell-based Therapies in the Treatment of Neonatal Hypoxic-ischemic Brain Injury

**DOI:** 10.1007/s12015-025-10905-9

**Published:** 2025-06-05

**Authors:** Paulina Gebala, Justyna Janowska, Joanna Sypecka

**Affiliations:** 1 Mossakowski Medical Research Institute, NeuroRepair Department, Polish Academy of Sciences, Pawinskiego 5, Warsaw, 02-106 Poland; 2 Doctoral School of Translational Medicine, Centre of Postgraduate Medical Education, Marymoncka 99/103, Warsaw, 01-813 Poland

**Keywords:** Stem cells, Neonatal brain, Hypoxic-ischemic injury, Neuroprotection, Neuroregeneration, Cell-based therapies

## Abstract

Neonatal hypoxia-ischemia is a leading cause of neonatal death in both developed and developing countries. The consequences of hypoxic-ischemic injury affect the rest of the child’s life, often resulting in intellectual or motor disability that persists into adulthood. To date, therapeutic hypothermia (TH) appears to be the only available intervention aimed at limiting brain injury and is recognized as the “gold standard” in neonatal intensive care. The basic mechanisms of neuroprotection achieved by temporal cooling involve the reduction of free radical activity, suppression of the inflammatory response after reperfusion and increased neuronal cell survival. However, the protective effects of hypothermia need to be enhanced by additional therapies that can enhance neuroprotection and support neuroregenerative processes. The components derived from the umbilical cord are thought to confer the above-mentioned beneficial effects. This review summarizes the clinical trials based on stem cell transplantation or umbilical cord milking and presents their effects when supported by official data. The great promise associated with the application of stem cells to neonates suffering from perinatal asphyxia is discussed in the context of the results of their clinical use.

**Graphical abstract:**

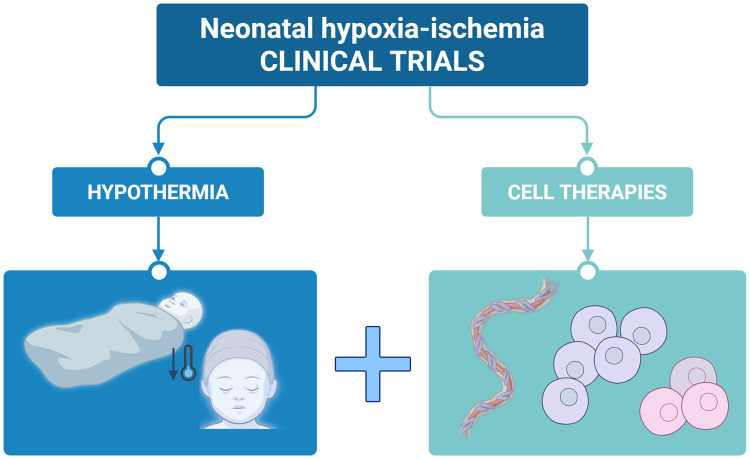

## Introduction

Neonatal asphyxia and its consequences, known as hypoxic-ischemic encephalopathy (HIE) represents a significant public health concern, contributing significantly to both neonatal mortality and morbidity worldwide [1–3]. According to the latest report of the World Health Organization (WHO) (January 2022), globally 2.4 million children died in the first month of life in 2020 (https://www.who.int/news-room/fact-sheets/detail/levels-and-trends-in-child-mortality-report-2021). The above mentioned report indicated perinatal asphyxia as one of the leading cause of neonatal morbidity and mortality in both the developed and developing countries. WHO estimates that more than 900,000 children died from birth asphyxia in 2014. The incidence of perinatal asphyxia is estimated for approximately two per 1000 births in high-resource settings, however the rate is up to 10 times higher in developing countries due to limited access to maternal and neonatal care [4–7]. The prognostic values indicate that 15–20% of asphyxiated newborns die in their neonatal period, while up to 25% of survivors will develop significant neurologic deficits [8, 9]. In case of neonates with severe HIE, they have a 60% chance of death, and nearly all survivors suffer from epilepsy and neurodevelopmental damage, such as cerebral palsy and intellectual incapacity [10, 11]. The resulting neonatal brain injury recognized as HIE, is one of the most fatal consequences of the experienced asphyxia [12–14].

A routine diagnostic method for assessing a newborn’s condition at birth is the Apgar score, which is a clinical tool used immediately after birth to assess a newborn’s physical condition and determine whether any urgent medical intervention is needed. When Apgar score estimated at 5 min after birth is below 7/10, perinatal asphyxia is suspected. If a five-minute Apgar score is determined as 0–3 points and additionally the pH measured in sample of the umbilical artery blood is lower than 7.0, severe birth asphyxia is diagnosed [15]. In presence of neonatal asphyxia and its consequences as like HIE, to describe the severity of injury within the first postnatal days the Sarnat staging criteria are applying, which distinguish 3 stages of neonatal HIE [16]:

- Stage 1 – tachycardia, hyperreflexia, hyperalertness, absence of seizures (mild).
- Stage 2 – bradycardia, hyperreflexia, miosis, seizures, lethargy, hypotonia with weak suck and Moro reflex (moderate).
- Stage 3 – hypothermia, stupor, flaccidity, decreased stretch reflexes, small to mid-position pupils that react poorly to light and absent Moro reflex (severe).

## Therapeutic Hypothermia (TH) - the First-line Neuroprotective Strategy

To date, the first-line strategy for limiting the extent of neonatal brain damage resulting from ischaemic-hypoxic injury induced by perinatal asphyxia is TH, which is recommended for term and, in some cases, preterm neonates. The temporary limited oxygen supply and trophic support initiate a cascade of events, which include among others excitotoxicity and oxidative stress resulting the increased rate of neuronal death and activation of glial cells, leading to induction of neuroinflammatory processes [17–20]. Lowering temperature in affected tissues allows to decrease metabolic rates in neural cells which are characterized by a high energy demand. Decrease in activity of metabolic processes means also reduction of oxygen requirements, thus promoting cell survival in HIE-associated pathophysiological conditions. Thus, it is also thought to mitigate the level and activity of reactive oxygen species (ROS), protecting tissues from further damage [21]. TH is recognized to be supportive for immunomodulatory processes, particularly during the reperfusion phase, when a massive production of pro-inflammatory factors occurs [22].

Most trials use whole-body hypothermia, while some trials use head cooling only. Clinical trials based on the beneficial effects of lowering the temperature of asphyxiated newborns date back to 1999, when the first patients were enrolled. From this first study, a number of clinical trials using different TH protocols were registered (Table 1).

**Table 1:** Clinical trials relating to therapeutic hypothermia in case of neonatal encephalopathy

NCT number/Country	Recruitment status	Initiation time of hypothermia	Duration of hypothermia	Apgar score	Age of children	Age eligible for study*	Number of participants	Reported outcomes	References
NCT00005772 United States	Completed (study completion-07.2010)	Within 6 h after birth	72-hour period	< 5 at 10 min	Up to 6 h	Up to 6 h	208	Reduced the risk of death and development of moderate to severe disability	[23–38]
NCT01092637 United Kingdom	Completed (study completion-07.2014)	Within 6 h after birth	72-hour period	< 5 at 10 min	Up to 6 h	72–87 months	280	Higher IQ, better cognitive development and lower degree of disability	[39–41]
NCT01793129 United States	Completed (study completion-12.2022)	Within 6 h after birth	72-hour period	< 5 at 10 min	Up to 6 h	33–35 weeks	168	Reduced the number of deaths, moderate or severe disability and serious adverse events	[42]
NCT00614744 United States	Completed (study completion-06.2016)	6–24 h of age	96-hour period	< 5 at 10 min	6–24 h	6–24 h	168	Reduced the risk of death rate and number of infants with mild, moderate and severe disability	[43, 44]
NCT02826941 United States	Completed (study completion-06.2004)	Within 6 h after birth	48-hour period	< 5 at 10 min	Up to 6 h	35 weeks and older	65	Relatively high incidence of side effects including lower heart rates, more frequent bradycardia and seizures	[45–48]
NCT00383305 United States, Canada, New Zealand, United Kingdom	Completed (study completion-09.2003)	Within 6 h after birth	72-hour period	< 5 at 10 min	Up to 6 h	1–6 h	235	Estimation of the early glycaemic profile in infants might be useful in predicting the risk of multiorgan dysfunction and response to TH, improved outcomes after TH were reported to be associated with diagnosed hyperglycaemia	[49–54]
NCT02683915 Egypt	Completed (study completion-09.2016)	Within 12 h after birth	72-hour period	Not reported	Up to 30 min to 24 h	Up to 30 min to 24 h	30	Less renal impairment, continuing kidney injury may persist in asphyxiated newborns despite improvement in serum creatinine	[55]
NCT03284528 Switzerland	Completed (study completion-01.2017)	Not reported	72-hour period	< 5 at 10 min	1–4 day old	1–4 day old	100	Not reported	[56]
NCT00890409 China	Completed (study completion-08.2005)	Within 12 h after birth	72-hour period	≤ 3 at 1 min and ≤ 5 at 5 min	Up to 6 h	Up to 6 h	256	Lower risk of severe neurodevelopmental disabilities, adverse events and death rates	[57]
NCT00817401 China	Completed (study completion-06.2008)	Up to 10 h after birth	72-hour period	< 5 at 5 min	Up to 10 h	1–10 h	100	Not reported	[58]
NCT02387385 Bangladesh India Sri Lanka	Completed (study completion-12.2020)	Within 6 h after birth	72-hour period	< 6 at 5 min (in babies born at hospital) or lack of cry by 5 min of age (for babies born at home)	Up to 6 h	Up to 6 h	408	No reduction of death or disability at 18 months when applied in low-income and middle-income countries, significantly increased death alone	[59–61]
NCT00620711 United States	Completed (study completion-07.2013)	Within 6 h after birth	72-hour period	0–3 at 1,5,10 min	30 min to 6 h	30 min to 6 h	4	No significant escalation in the number of disabilities, adverse events and mortality	[62]
NCT04621279 United States	Recruiting (study start-07.2023)	Within 6 h after birth	72-hour period	< 5 at 10 min	Up to 6 h	35 weeks and older	460	Not reported	[63]
NCT04176471 United States	Not yet recruiting (study start-05.2020)	Within 6 h after birth	72-hour period	< 5 at 10 min	Up to 6 h	Up to 6 h	68	Not reported	[64]
NCT01192776 United States	Terminated (study completion-03.2016)	Within 6 h after birth	72-hour period or 120-hour period	< 5 at 10 min	Up to 6 h	Up to 6 h	364	No reduction in death rates or disability at 18–22 months	[65–69]
NCT05581927 China	Withdrawn (study completion-12.2024)	Not reported	Not reported	< 5 at 10 min	0–24 h	0–24 h	0	Not reported	[70]
NCT01138176 India	Unknown (study completion-08.2011)	Not reported	72-hour period	< 5 at 5 min	Up to 24 h	Up to 24 h	35	Not reported	[71]

*age eligible for study– this term refers to the age of person who participate in a clinical study and the applicable eligibility criteria (consist of both inclusion and exclusion criteria) (Clinicaltrials.gov)

The clinical trial registered as (*NCT00005772*) among the first studies verifying the effectiveness of hypothermic approach. It was an interventional (parallel assignment), randomized in a phase III trial, in which the asphyxiated neonates with moderate to severe encephalopathy were randomized to a 72-hour period of total body cooling (cooling blanket, followed by slow rewarming), initiated within 6 h of birth. This trial was divided in two groups: the first group was focused on assessing the safety of maintaining an esophageal temperature within the range of 34.0–35.0 °C and second group aimed to evaluate both the safety and efficacy of maintaining a slightly lower esophageal temperature, specifically within the range of 33.0–34.0 °C. The neurodevelopmental outcome analyzed 18–22 months post-intervention revealed that whole-body cooling therapy reduced the risk of death and the development of moderate to severe disabilities in infants diagnosed with HIE [23–38].

In the cohort study (*NCT01092637*) the enrolled participants were divided in two groups. In the first group (i.e. experimental) newborns received standard intensive care plus moderate whole-body hypothermia within 6 h of birth to 72 h. The second group (placebo) consisted of newborns who only received the standard intensive care within 6 h of birth. The objective of the study was to conduct a follow-up assessment at 6–7 years of age on the children who took part in this trial, to investigate the effectiveness of the whole-body cooling therapy following perinatal asphyxia. The collected data indicated that the group of children who participated in experimental group showed a higher intelligence quotient (IQ), had better cognitive development and showed a lower degree of disability [39–41].

The next completed, interventional (parallel assignment), randomized clinical trial (*NCT01793129*) on TH ended in December 2022. In the experimental group, the whole-body hypothermia (with a target esophageal temperature of 33.5 °C) was induced for 72 h, while control group was maintained for 72 h in normothermic conditions (with esophageal temperature at or near 37.0 °C). The main focus of this study was to assess the occurrence of death or the development of moderate to severe disability among participants at the corrected age of 18–22 months. In this study, the presence or absence of disability was determined using the standard Neonatal Research Network (NRN) interdisciplinary follow-up examination. The results obtained after 18–22 months of age indicated reduction in number of deaths, moderate or severe disability and serious adverse events in infants with HIE [42].

Another interventional (parallel assignment), randomized study (*NCT00614744*) was conducted on the group of 168 infants in age up to 6–24 h with signs of HIE. Participants were randomly assigned to receive either 96 h of whole-body hypothermia or to participate in a non-cooled control group. The goal of the study was to prove whether hypothermia would reduce mortality and disability in children at the age of 18–22 months [43, 44]. According to the published conclusions, there is as much as a 76% probability of reduction the of death rate and number of infants with mild, moderate and severe disability, if hypothermia was initiated within 6 to 24 h of birth.

Different protocol of application of TH to 65 newborns was used in interventional (parallel assignment), randomized in a phase II clinical trial (*NCT02826941*). In the treated group, the children’s heads and bodies were covered with plastic bags filled with ice wrapped in a washcloth for 2 h and then with a cooling blanket to reduce the rectal temperature to 33.0 °C ± 0.5 °C for 48 h. In the normothermic group of infants, rectal temperatures were maintained at 37.0 °C ± 0.5 °C in accordance with the standard protocol used in neonatal intensive care units (NICU). During the following 12 months after treatment, the outcomes were monitored using the Bayley II or Vineland tests [45–48]. The results of the study indicated a relatively high incidence of side effects including lower heart rates and more frequent bradycardia in cooled newborns. Seizures were also more common in the hypothermia group. However, hypothermic treatment turned out to be effective in modulating activity of selected chemokines (monocyte chemotactic protein-1 and interleukin-8), thus exerting the immunomodulatory effect.

Further research on hypothermia (*NCT00383305*) – an interventional (parallel assignment), randomized study was about application cool cap with mild whole body hypothermia. The study, known as CoolCap, was conducted between 1999 and 2002 in 25 perinatal centers in the UK, USA and New Zealand. The total number of 234 term infants with moderate to severe neonatal encephalopathy were enrolled into this study. Infants up to 6 h after birth were subjected to cooling application for 72 h. Neurodevelopment of children was estimated at 18 months of age [49–54]. The study indicated that estimation of the early glycaemic profile in infants with moderate to severe hypoxic-ischemic injury might be useful in predicting the risk of multiorgan dysfunction and response to TH. Improved outcomes after TH were reported to be associated with diagnosed hyperglycaemia.

The cooling cup was also used in another study, registered as clinical trial (*NCT02683915*). As many as thirty full term infants within 12 h after birth were enrolled to cooling group for 72 h using cooling caps, while neonates in control group were only provided with medical care. The treated newborns were cared for under an overhead radiant heater, which is servo-controlled on the basis of the skin temperature of the infant’s abdomen, which can be regulated to sustain the rectal temperature within the range of 33.5–34.5 °C [55]. The selected renal parameters were measured and it was shown that TH treatment resulted in less renal impairment, but continuing kidney injury may persist in asphyxiated newborns despite improvement in serum creatinine.

In the cohort study (*NCT03284528*) 100 participants were treated by whole-body hypothermia for 72 h. The research involved infants aged around 1–4 days with HIE. The aim of this retrospective study was to evaluate whether the placenta could serve as a biomarker for predicting neurodevelopmental outcome. Accordingly, the correlation between placental histology and neurodevelopment of asphyxia survivors at 18–24 months of age will be assessed [56].

The cooling cap with mild hypothermia was used also in an interventional (parallel assignment), randomized trial in a phase III (*NCT00890409*). The procedure of cooling head started up to 6 h after birth within infants with Apgar score ≤ 3 at 1 min and ≤ 5 at 5 min. In experimental group the rectal temperature was maintained at 34.5 °C to 35.0 °C, but in control group was maintained at 36.0–37.5 °C [57]. The study indicated, that therapy caused lower risk of severe neurodevelopmental disabilities, adverse events and death rates.

In the interventional (parallel assignment), randomized in a phase I and II clinical trial (*NCT00817401*) researchers proposed to induce TH up to 10 h after birth, not up to 6 h like it was in majority of other studies. The 100 participants with Apgar score < 5 at 5 min were divided into treated and control groups. In the former, each neonate was surrounded by cooling mattress. The rectal temperature goal was set at 33.5 °C. The latter, control group was provided by standard medical care [58].

The whole body cooling was applied also in the interventional (single group assignment), randomized study, known as Low and Middle-Income Countries Trial (HELIX, *NCT02387385*) and conducted between Aug 15, 2015 and Feb 15, 2019. The entirely 408 participants from India, Sri Lanka and Bangladesh were enrolled into the study (202 to the hypothermia group and 206 to the control group). The cooling device (Tecotherm) was used in experimental group of children with HIE up to 6 h after birth to 72 h with constant temperature 33.5 °C. The researchers analyzed weather TH reduced death or neurodisability at 18 months after neonatal encephalopathy [59–61]. The results of this broad analysis indicated that TH did not reduce the combined outcome of death or disability at 18 months when applied in low-income and middle-income countries, but significantly increased death alone.

The four infants with Apgar scores of 0–3 recorded at 1, 5, 10 min were included in a pilot study (*NCT00620711*), an interventional (single group assignment) in a phase I using a cooling cap device for 72 h. The researchers observed, that benefits from TH seen at 18 months continued to childhood did not show an escalation in the number of infants experiencing disabilities, adverse events and mortality [62].

In the recruiting, observational study (*NCT04621279*) the effectiveness of hypothermia will be studied to compare mild HIE versus normothermia on neurodevelopmental outcomes at 2 years of age. The planned principles of the clinical trial are based on either establishing the whole-body TH (33.5 °C ± 0.5 °C) for 72 h starting 6 h after birth for neonates with mild encephalopathy or maintaining standard normothermia (core temperature corresponding to 36.5 °C ± 1.0 °C) with usual medical care for first 72 h for neonates with mild encephalopathy [63].

The registered study (TIME Study, *NCT04176471*) is designed to enroll into an interventional (parallel assignment), randomized trial around 68 neonates diagnosed with mild HIE across 5 centers in California, USA (coordinated by Stanford University). Infants are randomly assigned to receive either therapeutic hypothermia, maintained at 33.5 °C ± 0.5 °C for a duration of 72 h followed by 6 h of rewarming or normothermia with targeted temperature management aimed at maintaining temperatures within the range of 36.5–37.3 °C for the same 72-hour period [64].

In the large terminated trial (*NCT01192776*), an interventional (factorial assignment), randomized, which ended in April 2023, there were four planned options of therapeutic hypothermia. The group of 364 children was divided into subgroups in respect to the applied therapeutic conditions. Accordingly, whole-body hypothermia using a cooling blanket was planned as follows: at 33.5 °C for 72 h, at 33.5 °C for 120 h, at 32.0 °C for 72 h and at 32.0 °C for 120 h. The study found that deeper cooling and longer cooling were not associated with a reduction in death or disability at 18–22 months. However, the trial was stopped before completion due to emerging safety concerns and the results of a futility analysis, which led to the decision not to continue the trial [65–69].

Another planned clinical trial was withdrawn for lack of participants (*NCT05581927*). In this interventional (parallel assignment), randomized study the researchers planned to use the modified the procedure of whole body hypothermia versus standard whole body hypothermia. There was no data about duration of TH [70].

The status of an interventional (parallel assignment), randomized study in a phase I and II (*NCT01138176*) is unknown. The mentioned trial was designed to assess the safety and effectiveness of whole body cooling to a rectal temperature of 33.5 °C using phase changing material in HIE [71].

Despite numerous completed clinical trials, the results of the therapies used often remain inconclusive. However, there is a consensus that TH for mild to moderate HIE applied as soon as 6 h after birth and lasting for 72 h is beneficial in reducing cerebral palsy, neurological disability or cognitive impairment [39]. Analysis of health care costs associated with neurodisability in perinatal asphyxia survivors aged 6–7 years (including 130 children in the United Kingdom: 63 in the control group, 67 in the total body hypothermia group) showed that they were reduced by about 40% in the group of children treated with hypothermic rescue [40].

Although TH might confer neuroprotection in the critical post-injury period, it unfortunately has several limitations. First of all, controlled temperature reduction requires dedicated equipment (such as cooling device, disposable esophageal probe/rectal temperature probe, cardiorespiratory monitor) and qualified staff, which can be difficult in hospitals in low-income countries and in out-of-hospital care [72–74]. The current recommendations for inclusion criteria for the use of TH is a gestational age of at least 36 weeks. The diagnosis of perinatal asphyxia in preterm infants is challenging because preterm infants have lower body tone than term infants. Preterm infants have underdeveloped body organs (including cerebral vasculature), which increases the possibility of serious side effects such as coagulopathy, arterial hypotension, seizures, respiratory complications, thrombocytopenia, hyperglycaemia or persistent metabolic acidosis, which can be lethal. However, premature infants can be treated with TH if supported by qualified medical assistance (e.g. inotropes and steroids for diagnosed hypotension) [75, 76]. However, it has been shown that TH applied less than 6 h after birth to newborns at 34 weeks’ gestation did not reduce death or disability evaluated at 18 to 22 months [77].

Reducing the extent of brain damage through the use of hypothermia must be supported by therapies with pro-neuroregenerative potential to promote the restoration of the physiological course of neurodevelopmental processes. In the case of perinatal asphyxia, the use of stem cells appears to be the most attractive and promising option.

### Stem Cell Transplantation - a Hope for Boosting Neuroprotection and Improving Neuroregeneration

Stem cell therapy holds promise as an emerging and innovative treatment for many central nervous system (CNS) disorders [78]. Stem cells are derived from different tissue types and are characterized by their ability to self-renew and multipotency. Various types of stem cells, including umbilical cord blood cells (UCBC), neural stem/progenitor cells (NSPC) embryonic stem (ES) cells, bone marrow stromal cells and induced pluripotent stem cells (iPS) were used in the research focused on the stem cell treatments for CNS disorders [79].

Given the complex pathophysiological changes in the developing brain resulting from multifactorial processes triggered by neonatal hypoxia-ischemia, stem cell properties may exert multidirectional beneficial effects. These include modulation of the local microenvironment by releasing active molecules (e.g. neurotrophins, mitogens, growth factors, enzyme inhibitors etc.) that help to maintain tissue homeostasis and make it permissive for physiological, endogenous repair processes. By releasing anti-inflammatory cytokines in a paracrine manner, stem cells can contribute to the immunomodulatory effect by promoting the polarization of microglia towards the anti-inflammatory (M2) phenotype. Finally, in a supportive microenvironment, stem cells can be directed towards commitment to neural progenitors, partially replacing cells that did not survive the hypoxic-ischemic event [80–82].

Human UCBC represents a valuable source of both stem and progenitor cells. Therapeutic potential of UCB-derived cells was evaluated in many laboratories using animal models of neonatal hypoxic-ischemic injury. In the majority of these preclinical investigations, UCBCs were administered systemically and a significant part of them demonstrated positive therapeutic effects [83]. It was proven that even a single infusion of UCBC suspension improve long-term behavioral outcomes in a rat model of neonatal hypoxic-ischemic injury [84]. The collection of UCBC at birth is very safe, resulting in huge amount of cells containing a diverse array of stem and progenitor cells. These cells have been shown to have beneficial effects on several types of neurological cells, including glial cells, neurons and those involved in maintaining the blood-brain barrier. Accumulating evidence from numerous studies indicate the neuroprotective and neuroreparative advantages associated with early UCBC therapy for the developing brain [84–87]. The regenerative effects are acquired due to paracrine release of factors like: insulin-like growth factor 1 (IGF-1), epidermal growth factor (EGF), vascular endothelial growth factor (VEGF), glial cell line-derived neurotrophic factor (GDNF), brain-derived neurotrophic factor (BDNF), nerve growth factor (NGF) and anti-inflammatory interleukines (e.g. IL-10, IL-4).

## Umbilical Cord Blood Collection and Banking

Human UCBC are a valuable cellular reservoir of hematopoietic stem cells (HSC). These are used in transplantation as an alternative to bone marrow or peripheral blood progenitor cells. The HSC transplantations are widely performed for treating hematopoietic diseases, such as leukemia [88–91]. The UCBC are accessible, and can be collected through a safe and non-invasive process. Importantly, UCBC can be cryopreserved for many years for use by future recipients [92, 93]. The limitation of using UCBC is its low cell volume, which can pose challenges for successful transplantation in adults [94]. Many studies are focusing on ways to speed up engraftment and reduce transplant-related mortality (TRM). The new option is a double cord transplantation, which utilizes cord blood units from two donors and has facilitated the wider use of UCBC in adults. Other approaches include in vitro cell expansion under xeno-free and physiologically normoxic conditions to enable their use in the clinic [90, 93, 95, 96].

The first step before collecting blood from UCBC is obtaining the informed consent and mothers must be tested for diseases, such as Hepatitis B and C, or HIV reactivity. Blood from the umbilical cord should be collected immediately after birth of the newborn, before delivery of the placenta [97]. Collection is performed using bag systems or syringe. The procedure is painless and simple, but there is a high risk of UCBC contamination [98, 99]. Maintaining optimal temperatures during transportation and storage is crucial before processing, as it can significantly impact cell viability. Various studies have highlighted the different temperature ranges (lowest 4 °C [100, 101], to the highest 15.0–25.0 °C [99]) recommended for sample storage and transport to Umbilical Cord Blood Units. The UCBC must be transported to the laboratory within 28–34 h under carefully monitored conditions. Before cryopreservation, the red blood cells (RBC) are removed from the UCBC [98, 99]. This step is essential, because most stem cells are found in the mononuclear cell fraction (MNC), which is the only component required for banking. Removing RBC, which make up over 50% of the collected blood, enhances stem cell recovery. Reducing the volume of UCBC benefits storage by saving space, minimizing the amount of dimethylsulfoxide (DMSO) used in cell products and reducing the cytotoxic effects associated with RBC thawing [102–104]. To improve stem cell viability, techniques such as density gradient separation and gelatin sedimentation are used [105].

In cryopreservation, extremely low temperatures are used to preserve the integrity of cells and tissues. Once frozen, cells and tissues remain stable, stored at or near liquid nitrogen temperatures (− 196 °C). Cryoprotectants are essential to ensure cell survival. Another method is vitrification, where the aqueous system solidifies without ice crystallization or growth [106]. Although UCBC processing methods are cost-effective, they incur higher banking costs and are more challenging to thaw. Properly washed and thawed platelet-depleted UCBC units yield higher total nucleated cells (TNC), CD34 + cells and advanced cell engraftment than RBC-containing units [107, 108].

## Ethical Concerns of Banking

In banking practice, legal and regulatory standards are very important. This is related to obtaining an informed consent, legal outline, personal data protection, rights related to health care, relations between recipients, patients, physicians etc [109]. One of the main concerns is that UCBC should not change routine obstetric or neonatal care practices, such as delayed cutting and clamping of the umbilical cord. The timing of clamping and cutting the umbilical cord became a point of contention during UCBC collection [110].

The units, where UCBC are stored can be divided into: public, private or hybrid banks.

1. Public Banking

The public banks collect and store the UCBC for allogeneic use. Units stored in public banks are cataloged, their registries can be accessed nationally and internationally. Processing fees are applied to offset part of the storage and administrative expenses. Majority of funding for public banks comes from contributions, government support or grants [111]. During storage, UCBC units are HLA-typed and their information is entered into an international registry. Cord blood units listed in international stem cell registries often provide access to rare HLA alleles that are difficult to locate in bone marrow repositories [112, 113]. The public banks must have a strict quality standards, including a minimum nucleated cell count and volume. Moreover, the collected UCBC must be free from contamination, and the newborn’s birth records must be documented to exclude the infectious diseases. A sample of the mother’s blood should also be provided, as well as the genetic and medical history of the family [114].

2. Private Banking

The private banks provide storage of UCBC for the child or family members. This type of banking requires parents to pay an collection fee [115]. Private banks require an upfront fee for the initial collection of cord blood, followed by annual fees for ongoing storage and management. As for-profit entities, these banks are generally subject to less stringent regulation compared to public banks [111]. These days private banks offer UCBC donation to siblings also. In these cases, cord blood from one healthy sibling is stored for the other. However, UCBC grafts are often unsuitable for autologous use. Autologous cord blood is generally not appropriate for treating genetic disorders as the HSCs in the cord blood have the same genetic mutations as the patient [116, 117].

3. Hybrid Banking

Hybrid banks combine the features of both public and private cord blood banks. While typically categorized as a public or private, these banks also provide services associated with the other model. For example, a public bank may offer a private storage option for a designated fee. Another variation of hybrid banking allows for cord blood donation specifically for a pre-matched relative of the donor, often referred to as a directed donor bank [111, 118].

The benefits of public or private UCBC banks depend on the patient’s specific needs. It is essential to consider the distinction between autologous and allogeneic UCBC. Since the stored blood contains the same genetic material as the donor, UCBC collected from a newborn are not suitable for treating cancers or genetic disorders in the same individual. Researchers and healthcare professionals play a critical role in providing parents with accurate information regarding the purpose and potential applications of banked UCBC, as this is a key ethical concern. Public UCBC banking is widely recommended for collecting umbilical cord blood for transplantation, therapeutic purposes and other medical uses. Public banks focus primarily on promoting allogeneic donations, a practice similar to the collection methods used by traditional blood banks [119, 120].

## Clinical Trials Using Umbilical Cord Derivatives

The clinical trials aimed at elucidating the utility of potential stem cells transplants as cure for the consequences of perinatal asphyxia are summarized in Table 2.

**Table 2:** Clinical trials relating to cells transplants for curing neonatal encephalopathy

NCT number/ Country	Recruitment status	Source of the cells	Administration	Schedule of administration	Apgar score	Age of children	Age eligible for study	Number of participants	Reported outcomes	References
NCT02256618 Japan	Completed (study completion-07.2019)	Autologous umbilical cord blood cells	Intravenously	3 doses infused at 12–24, 36–48 and 60–72 h after birth	≤ 5 at 10 min	Up to 24 h	Up to 24 h	6	No changes in physiological and peripheral blood parameters, no adverse events, neurofunctional development was normal in most infants without impairment, noted delayed cerebral palsy	[121]
NCT00593242 United States	Completed (study completion-01.2017)	Autologous umbilical cord blood cells	Intravenously	4 infusions of 1–5*10^7^ cells/kg with the first dose as soon as possible after birth and at 24, 48 and 72 postnatal hours	≤ 5 at 10 min	Up to 14 days	Up to 14 days	52	Reduction in death rates, seizures and an ECMO hospitalizations	[122]
NCT02612155 United States	Completed (study completion-08.2019)	Autologous umbilical cord blood cells	Intravenously	Up to 2 infusions of 2–5*10^7^/kg of autologous umbilical cord blood cells	≤ 5 at 5 min	Up to 6 h	Up to 6 h	35	Reduced mortality rates, seizures, serious adverse events and none of infant required ECMO	[123]
NCT01649648 Singapore	Completed (study completion-11.2015)	Autologous umbilical cord blood cells	Intravenously	Infusion during 3 days after birth	Not reported	1–3 days	1–3 days	2	Not reported	[124]
NCT02881970 France	Recruiting (study start-02.2020)	Autologous cord blood stem cells	Intravenously	5*10^7^/kg autologous mononuclear cells from umbilical cord blood	≤ 5 at 5 min	1–3 days	1–3 days	20	Not reported	[125]
NCT02434965 United States	Withdrawn (study not started) (study completion-01.2022)	Autologous cord blood cells/autologous human placental derived stem cells	Intravenously	1/3 of autologous cord blood cells infused within 24 h after birth (day 0), 1/3 on day 3, 1/3 on day 7 after birth; 1/2 autologous human placental derived stem cells infused on day 2 and 1/2 on day 8 after birth	≤ 5 at 10 min	1 min to 6 h	1 min to 6 h	0	Not reported	[126]
NCT02551003 China	Withdrawn (study completion-12.2016)	Autologous cord blood cells	Intravenously	Infusions in 3 doses: first dose after birth, second at 48 h, third at 72 h after birth	≤ 5 at 10 min	Up to 24 h	Up to 24 h	0	Not reported	[127]
NCT02287077 India	Completed (study completion-10.2016)	Autologous umbilical cord blood cells	Milking umbilical cord	3 times milking umbilical cord before clamping the cord	Not reported	Up to 1 min	Up to 1 min	101	Not reported	[128]
NCT00375908 United States	Completed (study completion-12.2008)	Umbilical venous blood	Not reported	Draw up to 20 ml of umbilical venous blood from infants	< 7 at 5 min	> 34 or < 34 gestational age	Child, adult, older adult	4	Not reported	[129]
NCT06427642 China	Recruiting (study start-04.2022)	Umbilical cord blood mononuclear cells	Intravenously	Infusion of umbilical cord blood mononuclear cells given within 24 h of being identified as a high-risk patient	Not reported	1–28 days	1–28 days	120	Not reported	[130]
NCT03635450 United States	Completed (study completion-12.2020)	HCT-MSC	Intravenously	Infusion of 2*10^6^/kg hCT-MSC in first cohort (3 patients) primary dose in the first 48 h; second cohort (3 patients) primary dose in the first 48 h and secondary dose after 2 months	Not reported	0–48 h	0–48 h	6	Developmental outcomes were in average to low-average developmental assessment standard scores	[131]
NCT04261335 Japan	Completed (study completion-12.2022)	CL2020 cells	Intravenously	1,5–15 millions of cells on 5–14 days after birth	≤ 5 at 10 min	4–14 days	4–14 days	9	Not reported	[132]

Accordingly, the autologous UCBC transplants were used in an interventional (single group assignment) study in a phase I (*NCT02256618*), which started in 2014 and was completed in 2019 in Japan. Children with signs of neonatal encephalopathy received intravenously 3 doses of autologous UCBC at 12–24, 36–48, and 60–72 h after the birth. The participants were followed for neurodevelopmental outcome up to 18 months [121]. At this latest time points of neurological examination, neurodevelopment was reported to be normal without any impairment in four of the six patients who received treatment, did not observe changes in physiological and peripheral blood parameters. No adverse events occurred, also delayed cerebral palsy occurred after treatment.

In a similar, interventional (single group assignment), non-randomized trial in a phase I (*NCT00593242*) conducted in USA between January 1, 2009 and June 5, 2012. Altogether 23 newborns with hypoxic-ischemic injury enrolled into the study were cooled to 33.5 °C for 72 h and received up to 4 intravenous infusions of volume-reduced autologous, noncryopreserved UCBC cells at 24, 48 and 72 postnatal hours. Doses contained 1–5*10^7^ cells/kg and a targeted dose volume was below 2 ml/kg body mass. The measured blood physiological parameters of patients between pre- and post-first infusion and post-third infusion suggest that autologous UCBC treatment is safe [122]. The study design proven that collection, preparation and infusion of fresh UCBC to newborns who experienced HIE is feasible, although requires extensive and well-coordinated cooperation of members of both the intensive care units and cord blood bank, who prepared the samples. Results showed, that therapy caused reduction in death rates, seizures and an ECMO hospitalization.

Another research concerning the infusions of UCBC was an interventional (parallel assignment), randomized trial in a phase II (*NCT02612155*), which was registered in 2015 in USA. Newborns with moderate to severe HIE (up to 160 participants), treated with a standard TH protocol, received up to two infusions (with a dose range of 2–5*10^7^ cells/kg per dose) of autologous nucleated cord blood cells after cooling. The number of doses will be finally determined depending on the amount of available cord blood cells. The placebo group received a mix of autologous cord blood red cells and plasma infusions. According to the update posted in May 2024, thirty five participants have been enrolled into the study (17 in a group of cell recipients and another 18 participants in a placebo group). Outcomes were measured at 22–26 months by assessment of functional neurodevelopment [123]. Results showed, that after performed therapy occurred reduction in mortality rates, decreased presence of seizures and serious adverse events. Moreover, none of infant required ECMO hospitalization.

The interventional (single group assignment) trial in a phase II (*NCT01649648*) stared in 2011 in National University Hospital, Singapore, tested the safety of infusions of autologous cord blood cells in term gestation newborns during HIE. The infants will undergo follow-up assessments to evaluate their neurodevelopmental outcomes (using e.g. Peabody tests, Bayley Scales of Infant Development) with a time frame of 1 month to 2 years, at multiple time points: 1 month, 4–6 months, 9–12 months and 18–24 months after birth. Results will be supported by brain MRI at 1–2 weeks and 4–6 months of age [124]. To date, no results of the study have been posted in the public databases.

An interventional (single group assignment) clinical trial study (*NCT02881970*, NEOSTEM) in a phase I and II, which started in 2017, is recruiting patients with HIE symptoms (the final number of participants has been established at 20). The primary objective of this research is to evaluate the safety and feasibility of using autologous cord blood stem cells as a curative treatment for neonatal HIE. Patients with symptoms of HIE will be treating with autologous mononuclear cord blood stem cells (5*10^7^/kg intravenously) [125].

The trial, which was eventually withdrawn to the unknown reason, was designed as an interventional (single-group assignment) trial in a phase II (*NCT02434965*), started in 2019 in New York Medical College. In this study researchers proposed to combine two experimental therapies such as: infusion of UCBC together with stem cells derived from placenta. Both types of cells were collected from infants and administered at intervals during the first week of life to patients with severe HIE. The autologous cells were infused in separate portions: half of the isolated cells were infused on day 2 and the other half on day 8 after birth. The autologous cord blood cells were administered in portions. One third of the collected cord blood was infused within the first 24 h (day 0), another third on day 3 and the final third one on day 7. This study was designed to determine the tolerability and feasibility of an experimental cure for severe HIE [126].

The next trial (*NCT02551003*) - an interventional (parallel assignment), randomized in a phase I and II - used a regimen of autologous cord blood cells and hypothermia. The interventional research was conducted to predict how the administration of autologous cord blood cells and hypothermia affect HIE symptoms. Autologous cord blood will be collected after birth and stored in Cord Blood Bank of hospital. In the experimental group, infants received 3 doses of autologous cord blood cell infusions on the day of birth, after 48 h and 72 h, together with hypothermia therapy for 72 h within 6 h of birth. The control group received only TH. After 18 months, the following outcomes were assessed [127]. According to the post published on clinical trial website in December 2023, the trial has been withdrawn.

Another type of therapeutic strategy, based on cord milking, was tested in an interventional (parallel assignment), randomized trial (*NCT02287077*). The aim of this trial, which started in 2014, was to test the effectiveness of cord milking (3 times before clamping the cord) in newborns who had experienced neonatal asphyxia. Total number of 101 participants were enrolled into this trial (50 in the group of patients receiving treatment and 51 control group). In the experimental group the cord was clamped after 3 times of milking, but in the control group the cord was clamped immediately. The researchers found that this operation could increase the level of stem cells and hemoglobin in the blood circulation [128]. Breathing and swallowing difficulties can be a significant obstacle to this type of therapy in children who have received a low number of Apgar points.

In another approach, the investigators from Johns Hopkins University aimed to conduct a proteomic analysis of umbilical venous blood obtained from neonates with brain injury and compare it with samples from gestational age-matched non-injured controls in observational case-control study (*NCT00375908*). The study started in 2015 and is planned to analyze protein content in umbilical blood (20 ml from each cord) taken from 450 infants. Identified specific proteins will be compared between control group of healthy children and those with the suspected brain injury [129].

A combination of TH and umbilical cord cell transplantation (*NCT06427642*), an interventional (parallel allocation), randomized trial is recruiting patients with high-risk symptoms of HIE, including bronchopulmonary dysplasia (BPD) or short bowel syndrome (SBS). How to prevent such diseases is a key research topic of this study. Children in the experimental group will undergo both TH for 72 h and intravenous infusions of mononuclear cord blood cells within 24 h of being identified as a high-risk patient. Children in the control group will only receive TH [130].

Infusions of allogenic human umbilical cord tissue-derived mesenchymal stromal cells (hCT-MSC) in newborns with HIE were tested in a prospective, open-label, interventional (parallel assignment), non-randomized trial in a phase I (*NCT03635450*). The hCT-MSCs were derived from umbilical cord tissue preserved in a public cord blood bank at Duke University Medical Center with the consent of the child’s mother [131]. Patients were subjected to routine NICU practice and cooled to 33.5 °C for the first 72 h after birth. 6 patients, who had signs of neonatal hypoxia-ischemia (a moderate encephalopathy was recognized in 4 infants while 2 infants were classified as having severe encephalopathy), received intravenously one or two infusions consisted of hCT-MSC: all received 1 dose of hCT-MSC during cooling and 2 received a second dose 2 months later. Each dose consisted of 2*10^6^ hCT-MSCs/kg with the verified cell viability. The developmental outcomes evaluated between 12 and 17 postnatal months were in average to low-average developmental assessment standard scores.

New type of cells are CL2020 cells (allogenic human multilineage-differentiating stress-enduring cell-Muse based product) used in the interventional (single group assignment) study in a phase I (*NCT04261335*, SHIELD trial), initiated in Nagoya University Hospital, Japan, in March 2020. This is the first clinical trial performed with use of CL2020 cells in neonates. Muse cells are a fraction of endogenous, non-tumourigenic cells that exhibits pluripotent-like stem cells features such as very low immunogeneity (due to expression of (HLA)-G antigen), an ability to self-renew and differentiation into each of the three germ layer cells. The cell transplantation will be proceeded by TH therapy with cooling to a body temperature of 33.0–34.0 °C for 72 h. Neonates in the low-dose and high-dose cohorts receive intravenously 1.5 and 15 million cells per dose, respectively, between 5 and 14 days of age, which consequently determined the scope of safety and tolerance during the development of children. The first outcomes were measured at 18 months of age [132]. The result of the study will be announced.

To sum up, various protocols od stem cell administration of the HIE patients were designed to test first of all the safety of cell-based therapies. Although low immunogenic, when infused to the sick newborns, they could evoke side effects such as rash, respiratory problems, hypotension or swelling around mouth and eyes, especially when used as allogeneic transplants. Usually the cell-based protocols are combined with standard hypothermia-based treatment for the first 72 h of life in order to limit the spreading of brain damage. The different types of stem cells (derived from either cord blood or cord tissue, CL2020), various doses (ranging usually within 10^6^ to 10^7^ per kg/body weight) and ways of administration (intravenous infusion, cord blood milking) are tested, followed by assessment of neurodevelopmental outcomes. To date however, the well documented and described results of the only few trials have been published. Nevertheless, the data obtained so far are promising and give cautious hope that stem cells will become the basis for effective therapies in the nearest future.

## Discussion

The main cause of neonatal hypoxic-ischemic injury is a HIE. One of the consequences is psychomotor disability, which affects the child’s entire life. One of the primary methods during hypoxic-ischemic injury is a TH. It is also associated with adverse outcomes beyond the neonatal period, including cerebral palsy, cognitive impairment, epilepsy and other neurological conditions that manifest themselves at varying degrees. According to the clinical trials and meta-analyses, TH treatment provides benefits for full-term newborns with moderate to severe HIE, including reduced mortality and improved developmental outcomes. A growing list of evidence confirms that TH, when applied according to the standardized protocols, is safe and can enhance the neurological prognosis for children affected by HIE [133, 134]. The main problem in evaluating the results of clinical trials is the lack of fully published trial results and completed long-term follow-up data. However, the routine use of whole-body hypothermia in neonates with mild HIE is questionable and it is suggested that it should only be considered in a randomized controlled trial until its safety and efficacy are well established. It is crucial to conduct an appropriate neurological assessment before initiating hypothermia treatment [135]. The ongoing follow-up after discharge from hospital through childhood is critical for optimizing systemic and neurodevelopmental outcomes, as it may present opportunities for further neuroprotective interventions to mitigate brain injury [136].

Although treatment with TH provides neuroprotection to some degree, it must be enhanced by additional therapies. Stem cell engraftment is recognized as the most promising one, as potent to confer further neuroprotection, enhancement of endogenous neuroreparative mechanism, as well as immunomodulation. Moreover, there is a relatively wide range of possibilities to choose the source of stem cells. The further clinical trials are needed to evaluate which type of stem cells is most effective in contradicting the consequences of neonatal hypoxic-ischemic brain injury and in boosting neuroregeneration. In the summarized clinical trials of stem cell therapies for perinatal asphyxia, UCBC have been used. This regimen offers the possibility of autologous engraftment, but even in the case of allogeneic transplantation, umbilical cord mesenchymal/stromal cells are recognized to have a low immunogenic potential. Several studies also suggest that the paracrine activity of UCBC differs from cells derived from adult sources such as bone marrow or adipose tissue [137–139]. In this context, human umbilical cord stem cells were shown to have a broader secretome composition and higher levels of selected neurotrophins (especially BDNF, Neurotrophin 3), growth factors (Leukemia inhibitory factor-LIF, GDNF, GDNF) and immunomodulatory molecules (among others IL-6 and IL-8).

Currently, the clinical application of UCBC, CL2020 and Wharton-Jelly-derived mesenchymal/stromal cells is considered. The future perspectives include use of decellularized therapies as extracellular vesicles (EVs) derived from various types of stem cells and loaded with cargo of therapeutic potential [140–142]. One of the main advantages of future clinical protocols based on EVs is opportunity of intranasal application, which is a desired option in care of children with the low Apgar score. However, the manufacturing, safety and effectiveness of EVs in neonatal brain injury have to be determined [143, 144].

The stem cells-based therapies hold a great promise to deal with the later phases of injury evoked by hypoxic-ischemic event, when the local tissue homeostasis is not yet restored and energy crisis continues [145, 146]. Similarly, there is growing recognition of the potential for direct support in the energy crisis induced by hypoxic-ischemic conditions. As the mitochondrial network is known to be severely compromised in minutes, hours, days and even weeks after injury, the mesenchymal/stromal cells could provide direct mitochondrial transfer to support survival of neural cells in the fragile developing brain [147].

Cell-based protocols are typically used alongside with standard hypothermia treatment during the first 72 h of life to help contain the spread of brain injury. The results indicated that the primary mechanism of action for these treatments involves the secretion of neurotrophic and anti-inflammatory cytokines that support neurogenesis and tissue repair [148]. Additional research is needed to evaluate the therapeutic efficacy of MSCs for neurological conditions, such as perinatal asphyxia. This is a promising pathway for cell-based therapies to treat neurological disorders [149]. Intravenously administered CL2020 are being tested in clinical trials for the treatment of HIE and stroke. The studies may provide insights into the potential of stem cell-based therapies for neurological disease treatment [148]. Other approach to deliver umbilical cord-residing stem cells is milking before clamping the cord [128]. However, this strategy has some serious limitations, such as the lack of sucking and swallowing reflexes in neonates with low Apgar scores. Umbilical cord milking, or delayed cord clamping, is a procedure involving placental transfusion. It increases the volume of a newborn’s blood by 20–30%, while providing stem cells from the cord blood. However, the procedure has several limitations. One is the possibility of polycythaemia and hyperbilirubinaemia, caused by the increased blood volume. Furthermore, there is a high risk of early cord clamping in non-vigorous, asphyxiated newborns, which makes placental transfusion impossible. Additionally, preterm newborns who could potentially benefit from stem cell transfer are at risk of intraventricular haemorrhage if they are less than 28 weeks gestational age. Nevertheless, the conclusions of the first clinical trial to date suggest that umbilical cord milking appears to be safe and feasible with no apparent adverse effects, and that cord milking thus milking the umbilical cord before clamping may be a valuable and cost-effective practice to improve outcomes in non-vigorous infants [150].

**Fig. 1:**
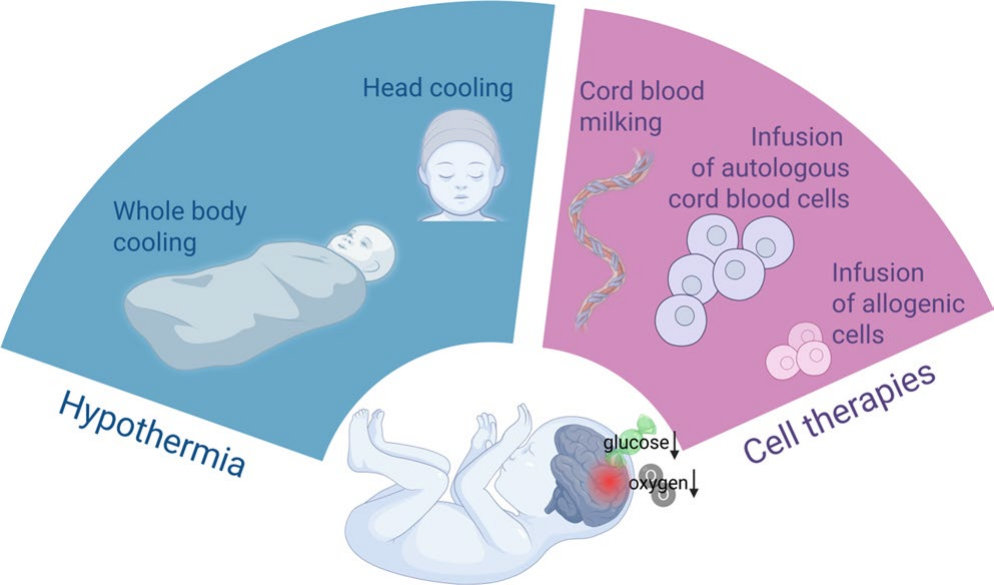
Clinical approach to deal with consequences of neonatal hypoxic-ischemic brain injury. Therapeutic hypothermia (head cooling vs. whole body cooling) is aimed at limiting the spread of injury, while stem cells are used to boost neuroprotection and neuroregeneration in the neonatal, injured brain

In conclusion, the nature of neonatal brain injury, such as perinatal asphyxia, poses significant challenges in the development of effective treatment strategies for these infants due to the need to implement standard procedures in the NICU, as well as the overall poor condition of the asphyxiated newborns. However, potential neuroprotective and neuroregenerative therapies have shown promise in preclinical studies and in some clinical trials, therefore combination of TH with simultaneous use of stem cells to boost neuroprotection and neuroregeneration seems to be promising therapeutic strategy to deal with fatal consequences of perinatal asphyxia (Fig. 1). With continued translational research, these approaches may help alleviate the significant burden of brain injury and disability associated with neonatal hypoxic-ischemic brain injury [151]. Treatment with stem cells or their derivatives, such as preconditioned (e.g. by hypoxic conditions) or genetically modified EVs to enhance their pro-regenerative potential, now appears to be the most optimal therapy to address many of the lethal consequences of neonatal hypoxic-ischemic brain injury and potentially improve long-term neurological outcomes.

## Data Availability

All data pertaining to this manuscript are included within the article.
